# Outcomes and prognosis of postpartum hemorrhage according to management protocol: an 11-year retrospective study from two referral centers

**DOI:** 10.1186/s13017-024-00556-5

**Published:** 2024-08-01

**Authors:** Ye Won Jung, Jin Kim, Won Kyo Shin, Soo Youn Song, Jae Sung Choi, Suk Hwan Hyun, Young Bok Ko, Mina Lee, Byung Hun Kang, Bo Young Kim, Jin Hong Min, Yong Nam In, Sang Min Jung, Se Kwang Oh, Heon Jong Yoo

**Affiliations:** 1 Department of Obstetrics & Gynecology, Chungnam National University Sejong Hospital, Sejong, Republic of Korea; 2 Department of Public Health, Chungnam National University School of Medicine, Daejeon, Republic of Korea; 3 Department of Obstetrics & Gynecology, Chungnam National University Hospital, Daejeon, Republic of Korea; 4 Department of Emergency Medicine, Chungnam National University Sejong Hospital, Sejong, Republic of Korea; 5 Chungnam National University School of Medicine, Daejeon, Republic of Korea

**Keywords:** Postpartum Hemorrhage, Lactic acid, Hemorrhagic shock, Uterine Atony, Hysterectomy, Disseminated intravascular coagulation, Uterine artery embolization

## Abstract

**Background:**

No standard treatment guidelines have been established for postpartum hemorrhage (PPH). We aimed to assess the differences in outcomes and prognoses between patients with PPH who underwent surgical and non-surgical treatment.

**Methods:**

This retrospective study included 230 patients diagnosed with PPH at two referral hospitals between August 2013 and October 2023. The patients were divided into non-surgical (group 1, *n* = 159) and surgical intervention groups (group 2, *n* = 71). A subgroup analysis was performed by dividing the surgical intervention group into immediate (*n* = 45) and delayed surgical intervention groups (*n* = 26).

**Results:**

Initial lactic acid levels and shock index were significantly higher in group 2 (2.85 ± 1.37 vs. 4.54 ± 3.63 mmol/L, *p* = 0.001, and 0.83 ± 0.26 vs. 1.10 ± 0.51, *p* < 0.001, respectively). Conversely, initial heart rate and body temperature were significantly lower in group 2 (92.5 ± 21.0 vs. 109.0 ± 28.1 beat/min, *p* < 0.001, and 37.3 ± 0.8 °C vs. 37.0 ± 0.9 °C, *p* = 0.011, respectively). Logistic regression analysis identified low initial body temperature, high lactic acid level, and shock index as independent predictors of surgical intervention (*p* = 0.029, *p* = 0.027, and *p* = 0.049, respectively). Regarding the causes of PPH, tone was significantly more prevalent in group 1 (57.2% vs. 35.2%, *p* = 0.002), whereas trauma was significantly more prevalent in group 2 (24.5% vs. 39.4%, *p* = 0.030). Group 2 had worse overall outcomes and prognoses than group 1. The subgroup analysis showed significantly higher rates of uterine atony combined with other causes, hysterectomy, and disseminated intravascular coagulopathy in the delayed surgical intervention group than the immediate surgical intervention group (42.2% vs. 69.2%, *p* = 0.027; 51.1% vs. 73.1%, *p* = 0.049; and 17.8% vs. 46.2%, *p* = 0.018, respectively).

**Conclusions:**

Patients with PPH presenting with increased lactic acid levels and shock index and decreased body temperature may be surgical candidates. Additionally, immediate surgical intervention in patients with uterine atony combined with other causes of PPH could improve prognosis and reduce postoperative complications.

## Background

Postpartum hemorrhage (PPH) is a serious obstetric complication that accounts for 27% of maternal deaths worldwide [1]. It is the fourth leading cause of maternal mortality in the United States and the leading cause of maternal mortality and morbidity worldwide [2]. PPH can be divided into two types: primary PPH, which occurs within 24 h (h) of delivery, and secondary PPH, which occurs between 24 h and 12 weeks after delivery. Currently, there is no consistent definition of PPH across countries; blood loss exceeding 500 mL after vaginal delivery or exceeding 1,000 mL after cesarean section has been defined as PPH by the Society of Obstetricians and Gynecologists of Canada (SOGC) [3], whereas the World Health Organization (WHO) defines PPH as blood loss exceeding 500 mL, regardless of the mode of delivery [4]. In 2017, the American College of Obstetricians and Gynecologists (ACOG) redefined PPH as 1,000 mL of blood loss accompanied by signs or symptoms of hypovolemia, regardless of the route of delivery [5]. PPH is the result of several factors that can occur alone or in combination, such as uterine atony, retained placental tissue, trauma including genital tract injuries, and coagulation dysfunction (the “4 Ts”: tone, tissue, trauma, and thrombin) [6]. Uterine atony is responsible for most (75%) cases of PPH [7].

When PPH occurs, the treatment method is chosen based on the amount of bleeding, the patient’s vital signs, and the cause of the bleeding. Unless the condition is life-threatening, conservative treatment is usually administered before peripartum hysterectomy to preserve fertility. The first-line treatments for PPH include uterine massage, manual removal of residual placental tissue, bimanual compression maneuvers, gauze packing, application of uterotonics and hemostasis, and volume replacement. If unsuccessful, second-line treatments for PPH should be performed, such as uterine sandwich (B-Lynch suture and Bakri balloon tamponade [BBT]), uterine arterial ligation, and uterine arterial embolization (UAE) [8, 9]. Among these techniques, BBT has been recommended by the ACOG and the WHO as a second-line conservative treatment for PPH [10], and UAE has been proposed as a standard treatment option for PPH because of its low invasiveness and high success rate [11]. An appropriate second-line procedure may achieve hemostasis for intractable hemorrhage and prevent the need for more severe surgical procedures, including peripartum hysterectomy. Peripartum hysterectomy is usually performed in patients with severe bleeding that cannot be conservatively controlled. However, a management method called resuscitative endovascular balloon occlusion of the aorta (REBOA) was introduced in 2008 to obviate the need for peripartum hysterectomy and preserve the uterus in life-threatening cases of PPH with blood loss reaching 40% [12, 13]. In addition, REBOA is increasingly used to treat placenta accreta spectrum disorders [14].

Choosing an appropriate initial treatment method is important because it can make a difference between the life and death of a patient. However, to date, no optimal approach has been established to treat PPH with a 100% success rate, making the decision to perform hysterectomy difficult, particularly in young women and women of low parity [12–14]. Most existing studies have investigated the risk factors and prognosis of peripartum hysterectomy for PPH by dividing patients into groups that underwent peripartum hysterectomy and those that did not [15–17]. However, few studies have analyzed cases in which surgery was performed after the failure of second-line treatments such as BBT or UAE.

Therefore, this study investigated the causes of PPH and the differences in outcomes between surgical and non-surgical intervention groups. The surgical intervention group was further divided into two subgroups: immediate surgery and delayed surgery after the failure of BBT or UAE. This study aimed to assess the differences in outcomes between patients with PPH who underwent non-surgical, surgical, and surgical treatments after failure of non-surgical interventions.

## Methods

This retrospective study was conducted between August 2013 and October 2023 in the department of Obstetrics and Gynecology of Chungnam National University Hospital and Chungnam National University Sejong Hospital, which are tertiary referral centers for high-risk pregnancies. This study was approved by the Ethics Committee of Chungnam National University Sejong Hospital (IRP No. 2023-12-004). PPH was defined as blood loss exceeding 500 mL, regardless of the mode of delivery (vaginal or cesarean section), according to the WHO definition of PPH. Cases of primary PPH occurring within 24 h of delivery and secondary PPH occurring between 24 h and 12 weeks after delivery were included.

**Fig. 1:**
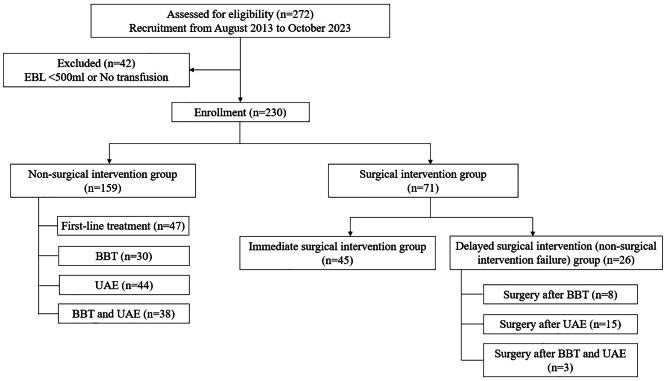
Flowchart of the study participant selection. EBL = estimated blood loss, BBT = Bakri balloon tamponade, UAE = uterine arterial embolization

Figure 1 shows the selection of the participants. Women diagnosed with PPH were included in the study. Of the 272 recruited patients, 42 were excluded because their estimated blood loss (EBL) was less than 500 mL, or no blood transfusion was performed. The remaining 230 patients were divided into two groups: non-surgical intervention (group 1, *n* = 159) and surgical intervention (group 2, *n* = 71). The surgical intervention group was further divided into two groups for subgroup analysis: immediate surgical intervention (*n* = 45) and delayed surgical intervention after the failure of non-surgical intervention (*n* = 26). First-line treatments included uterotonics, hemostasis, uterine massage, manual removal of residual placental tissue, gauze packing, and bimanual compression maneuvers. Second-line treatments included BBT and UAE. Surgical interventions included B-Lynch suturing, uterine artery ligation, surgical bleeding control or laceration repair, surgical placental removal, and hysterectomy. Failure of non-surgical intervention refers to the failure of second-line treatment.

The decision to perform surgery was made according to the institutional criteria. When patients with PPH visited the hospital, only first-line treatment was administered if their vital signs were stable, EBL was less than 1 L, or vaginal bleeding required 1–2 pads/h. If the EBL was 1–2 L or more than 2 pads/h, the second-line treatment was administered simultaneously with the first-line treatment. Surgery was performed immediately if the EBL exceeded 2 L and there was clinically severe and persistent uncontrollable hemorrhage or unstable vital signs.

The baseline obstetric characteristics of women with PPH, such as age, body mass index (BMI), parity, singleton or multiple pregnancies, gestational age at delivery, type of delivery (vaginal delivery vs. cesarean section), delivery location (inborn vs. outborn), baby weight, and previous uterine surgery (including a history of cesarean section or myomectomy), were obtained from the records. The time of PPH onset (< 24 h after delivery, or > 24 h after delivery up to 12 weeks), initial laboratory information, and initial vital signs were obtained from the records. Initial laboratory tests included white blood cell (WBC) count, hemoglobin, hematocrit (Hct), platelets, and lactic acid level. The initial vital signs included systolic blood pressure (BP), diastolic BP, heart rate, body temperature, and shock index (SI). The SI was calculated as heart rate divided by systolic BP.

The causes of PPH were classified according to the 4 Ts. “Tone” includes uterine atony, and “trauma” includes uterine wall rupture and genital tract injury. “Tissue” includes retained placenta and clots and abnormal placentation. “Thrombin” includes placental abruption, pre-eclampsia, and coagulation abnormalities. Abnormal placentation includes placenta previa and placenta accreta syndrome; genital tract injuries, including perineal, cervical, and vaginal lacerations; and extrauterine bleeding, including bleeding in the abdominal wall, intraperitoneal cavity, and surrounding organs.

Data on PPH and morbidity outcomes were also analyzed. The survey items were total EBL, total number of blood transfusion packs, mean hospital stay, intensive care unit (ICU) admission rate, mortality, and complications. Total EBL was estimated by measuring the weight of the blood-soaked pad, the weight of the gauze used before and after the procedure, and the amount of blood contained in the irrigation bottle used during surgery. One milliliter of blood, weighing approximately 1 g, was used. Blood transfusions were calculated as the number of red blood cell (RBC), fresh frozen plasma (FFP), and platelet transfusion packs. Complications included disseminated intravascular coagulopathy (DIC), fever, hypertension, wound infection, acute renal failure (ARF), pulmonary complications such as pulmonary edema and pulmonary effusion, cardiac complications such as heart failure and arrhythmia, cerebral complications such as cerebral hemorrhage, stroke, and headache, deep vein thrombosis (DVT), and Sheehan syndrome. In the subgroup analysis, we further investigated the following outcomes: time from decision to transfer to the start of surgery, hysterectomy rates, and surgical complications, including bladder injury, ureteral injury, and intestinal complications.

### Statistical analysis

Quantitative variables were described as mean ± standard deviation. Qualitative variables were described as frequencies (n) and proportions (%). An independent-samples t-test was used to compare the two groups. Logistic regression analysis was performed on the variables that showed significant differences ​​in the t-test. Statistical significance was defined as a *p*-value < 0.05. The statistical program SPSS (IBM SPSS Version 22.0) was used for analysis.

## Results

The obstetric and baseline characteristics of the patients with PPH are shown in Table 1. Age (33.2 ± 4.0 vs. 33.6 ± 4.2 years, *p* = 0.189), BMI (24.2 ± 3.0 vs. 24.8 ± 4.3 kg/m^2^, *p* = 0.322), and parity (91; 57.2% vs. 33; 46.5%, *p* = 0.132) showed no significant differences between the groups. Patients in group 2 exhibited a significantly higher initial lactic acid level (4.54 ± 3.63 vs. 2.85 ± 1.37 mmol/L, *p* = 0.001), initial heart rate (109.0 ± 28.1 vs. 92.5 ± 21.0 beat/min, *p* < 0.001), and SI (1.10 ± 0.51 vs. 0.83 ± 0.26, *p* < 0.001) than group 1. Conversely, initial systolic BP (107.4 ± 25.9 vs. 115.0 ± 19.3 mmHg, *p* = 0.029), and initial blood temperature (37.0 ± 0.9 vs. 37.3 ± 0.8 °C, *p* = 0.011) were significantly lower in group 2.

**Table 1:** Obstetric and baseline characteristics of patients with postpartum hemorrhage (*n* = 230)

Variable	Group 1 (*n* = 159)	Group 2 (*n* = 71)	*P*-value
Age (year)	33.2 ± 4.0	33.6 ± 4.2	0.433
BMI (kg/m^2^)	24.2 ± 3.0	24.8 ± 4.3	0.322
Parity (Primipara: Multipara)	91:68 (57.2:42.8)	33:38 (46.5:53.5)	0.132
Number of fetuses (Singleton: Multiple)	148:11 (93.1:6.9)	69:2 (97.2:2.8)	0.148
Gestational age (Fullterm: Preterm)	153:6 (96.2:3.8)	69:2 (97.2:2.8)	0.716
Type of Delivery (Vaginal delivery: Cesarean section)	98:61 (61.6:38.4)	40:31 (56.3:43.7)	0.451
Delivery location (Inborn: Outborn)	20:139 (11.9:86.8)	3:68 (4.2:95.8)	0.028
Baby weight (kg)	3.2 ± 0.4	3.2 ± 0.7	0.765
Previous uterine surgery	18 (11.3)	9 (12.7)	0.874
PPH onset (Primary: Secondary)	119:40 (74.8:25.2)	58:13 (81.7:18.3)	0.227
Initial Blood test			
WBC (10^3^/L)	17.5 ± 6.9	18.5 ± 7.6	0.294
Hb (g/dL)	9.4 ± 2.2	9.1 ± 2.6	0.398
Hct (%)	27.8 ± 6.5	27.1 ± 7.0	0.464
Platelet (K)	187.2 ± 87.8	164.1 ± 88.7	0.068
Lactic acid (mmol/L)	2.85 ± 1.37	4.54 ± 3.63	0.001
Initial Vital sign			
Systolic BP (mmHg)	115.0 ± 19.3	107.4 ± 25.9	0.029
Diastolic BP (mmHg)	69.0 ± 16.4	64.7 ± 18.3	0.074
Heart rate (beat/min)	92.5 ± 21.0	109.0 ± 28.1	< 0.001
Body temperature (°C)	37.3 ± 0.8	37.0 ± 0.9	0.011
Shock index	0.83 ± 0.26	1.10 ± 0.51	< 0.001

All data presented as mean ± standard deviation or number (%).

Group 1, non-surgical intervention group; Group 2, surgical intervention group; BMI, body mass index; PPH, postpartum hemorrhage; Hb, hemoglobin; Hct, hematocrit; BP, blood pressure; Shock index, heart rate / systolic blood pressure.

The causes of PPH in all patients are shown in Table 2. Among the sole causes of PPH, uterine atony accounted for the highest proportion at 47.8%. The proportions of trauma and tissue as the sole causes were similar, at 13% and 12.2%, respectively. A combination of atony and other causes was observed in 52 patients (22.6%). Uterine atony thus accounted for 70.4% of all PPH cases. Thrombin-related causes did not exist alone but were combined with other causes, accounting for six cases (2.6%).

**Table 2:** Causes of postpartum hemorrhage (*n* = 230)

Cause of postpartum hemorrhage	n (%)
Tone	110 (47.8)
Uterine atony	
Trauma	30 (13.0)
Uterine rupture Genital tract injury	
Tissue	28 (12.2)
Retained placenta Abnormal placentation	
Multifactorial	
Tone + Trauma	34 (14.8)
Tone + Tissue	15 (6.6)
Tone + Trauma + Tissue	3 (1.3)
Trauma + Tissue	4 (1.7)
Thrombin (placental abruption or preeclampsia or existing coagulopathy) ± tone ± trauma ± tissue	6 (2.6)
Total	230 (100)

Data are presented as number (%).

The outcomes of PPH are presented in Table 3. Uterine atony was a significantly more common cause of PPH in group 1 (91 cases, 57.2%) than in group 2 (25 cases, 35.2%; *p* = 0.002). Conversely, trauma was a significantly more frequent cause of PPH in group 2 (39 cases, 24.5%) than in group 1 (28 cases, 39.4%; *p* = 0.030). Patients in group 2 experienced significantly worse outcomes than those in group 1. They also had higher total EBL (*p* < 0.001), total blood transfusion requirements (*p* < 0.001), mean hospital stay (*p* < 0.001), and ICU admission rates (*p* < 0.001). In addition, the mortality rate in group 2 was significantly higher (*p* = 0.016). All investigated complication rates were also higher in group 2, including those of DIC (*p* < 0.001), fever (*p* = 0.024), hypertension (*p* = 0.009), wound infection (*p* = 0.033), ARF (*p* < 0.001), pulmonary complications (*p* < 0.001), cardiac complications (*p* < 0.001), cerebral complications (*p* = 0.002), and Sheehan syndrome (*p* = 0.002).

**Table 3:** Outcomes of postpartum hemorrhage (*n* = 230)

Variables	Group 1 (*n* = 159)	Group 2 (*n* = 71)	*P*-value
**Cause of PPH**			
Tone	91 (57.2)	25 (35.2)	0.002
Trauma	39 (24.5)	28 (39.4)	0.030
Tissue	31 (19.5)	21 (29.6)	0.112
Tone + other causes	32 (20.1)	20 (28.2)	0.201
**Total EBL (mL)**			< 0.001
>500 ─ <1000	85 (53.5)	12 (26.7)	
≥1000 ─ <2000	64 (40.3)	10 (22.2)	
≥2000	10 (6.3)	23 (51.1)	
**Blood transfusion**			
RBC (pack)	4.2 ± 3.0	13.4 ± 18.2	< 0.001
FFP (pack)	2.6 ± 2.9	11.7 ± 23.4	< 0.001
Platelets (pack)	1.0 ± 3.3	17.3 ± 31.2	< 0.001
Mean hospital days	4.1 ± 1.7	8.7 ± 10.3	< 0.001
ICU admission rate	6 (3.8)	17 (37.8)	< 0.001
Mortality	0	2 (4.4)	0.016
**Complications**			
DIC	14 (8.8)	19 (42.2)	< 0.001
Fever	19 (11.9)	10 (22.2)	0.024
Hypertension	2 (1.3)	1 (2.2)	0.009
Wound infection	0	2 (4.4)	0.033
ARF	0	3 (6.7)	< 0.001
Pulmonary complications	4 (2.5)	11 (24.4)	< 0.001
Cardiac complications	2 (1.3)	2 (4.4)	0.001
Cerebral complications	2 (1.3)	3 (6.7)	0.002
DVT	1 (0.6)	2 (4.4)	0.113
Sheehan syndrome	0	3 (6.7)	0.002

All data presented as mean ± standard deviation or number (%).

Group 1, non-surgical intervention group; Group 2, surgical intervention group; PPH, postpartum hemorrhage; EBL, estimated blood loss; RBC, red blood cell; FFP, fresh frozen plasma; ICU, intensive care unit; DIC, disseminated intravascular coagulation; ARF, acute renal failure; DVT, deep vein thrombosis.

Table 4 presents the results of the analysis investigating the influence of the initial laboratory and vital sign parameters that differed significantly between groups 1 and 2 on the decision-making process for surgical intervention in patients with PPH. Univariate regression analysis revealed that the initial systolic BP, heart rate, body temperature, lactic acid level, and SI were significantly associated with surgical intervention for PPH (*p* = 0.016, *p* < 0.001, *p* = 0.012, *p* < 0.001, and *p* < 0.001, respectively). Multivariate regression analysis identified initial body temperature (odds ratio [OR], 0.637; 95% confidence interval [CI], 0.424–0.956; *p* = 0.029), initial lactic acid level (OR, 1.253; 95% CI, 1.026–1.530; *p* = 0.027), and initial SI (OR, 180.54; 95% CI, 1.018-32,011.754; *p* = 0.049) as significant predictors of surgical intervention for PPH.

**Table 4:** Unadjusted and adjusted results of logistic regression analysis predicting surgery of postpartum hemorrhage (*n* = 230)

	Unadjusted		Adjusted	
Predictor	OR (95% CI)	*p*-value	OR (95% CI)	*p*-value
Initial systolic BP	0.984 (0.971─0.997)	0.016	1.034 (0.986─1.083)	0.166
Initial HR	1.029 (1.016─1.042)	< 0.001	0.977 (0.931─1.025)	0.338
Initial BT	0.645 (0.457─0.909)	0.012	0.637 (0.424─0.956)	0.029
Initial LA	1.375 (1.156─1.636)	< 0.001	1.253 (1.026─1.530)	0.027
SI	9.941 (3.825─25.834)	< 0.001	180.540 (1.018─32011.754)	0.049

Nagelkerke R^2^ = 0.296

OR, odds ratio; CI, confidence interval; BP, blood pressure; HR, heart rate: BT, body temperature; LA, lactic acid; SI, Shock index (heart rate / systolic blood pressure)

A subgroup analysis was conducted by dividing the surgical intervention group into two subgroups: patients who underwent immediate surgery and those who underwent delayed surgery following unsuccessful non-surgical management (Table 5). The subgroup analysis revealed a significantly higher rate of uterine atony combined with other causes of PPH in the delayed surgery group than in the immediate surgery group (8 cases; 17.8% vs. 12 cases; 46.2%, *p* = 0.018). Additionally, the delayed surgery group had significantly higher rates of hysterectomy (23 cases; 51.1% vs. 19 cases; 73.1%, *p* = 0.049) and DIC (19 cases; 42.2% vs. 18 cases; 69.2%, *p* = 0.027).

**Table 5:** Subgroup analysis of surgical intervention group (Group 2) (*n* = 71)

Variables	Immediate surgical intervention group (*n* = 45)	Delayed surgical intervention (non-surgical intervention failure) group (*n* = 26)	*P*-value
Initial Lab, vital sign			
Lactic acid (mmol/L)	4.46 ± 3.95	4.68 ± 3.06	0.813
Systolic BP (mmHg)	111.4 ± 25.9	100.4 ± 24.9	0.083
Heart rate (beat/min)	112.6 ± 30.4	102.7 ± 22.9	0.152
Body temperature (°C)	36.9 ± 0.9	37.1 ± 0.9	0.267
Shock index	1.11 ± 0.6	1.10 ± 0.4	0.943
From transfer to Operation time (min)	204.4 ± 248.4	305.7 ± 235.6	0.106
Cause of PPH			
Tone	17 (37.8)	8 (30.8)	0.558
Trauma	16 (35.6)	12 (46.2)	0.386
Tissue	15 (33.3)	6 (23.1)	0.369
Tone + other causes	8 (17.8)	12 (46.2)	0.018
Total EBL (mL)			0.152
>500 ─ <1000	12 (26.7)	4 (15.4)	
≥1000 ─ <2000	10 (22.2)	4 (15.4)	
≥2000	23 (51.1)	18 (69.2)	
Blood transfusion			
RBC (pack)	13.5 ± 18.1	13.8 ± 11.6	0.945
FFP (pack)	11.7 ± 23.4	12.3 ± 10.9	0.913
Platelets (pack)	17.3 ± 31.2	12.2 ± 15.7	0.445
Mean hospital days	8.7 ± 10.3	10.4 ± 13.6	0.546
ICU admission rate	17 (37.8)	12 (46.2)	0.496
Mortality	2 (4.4)	0	0.160
Hysterectomy done	23 (51.1)	19 (73.1)	0.049
Complications			
DIC	19 (42.2)	18 (69.2)	0.027
Fever	10 (22.2)	8 (30.8)	0.432
Hypertension	1 (2.2)	3 (11.5)	0.178
Wound infection	2 (4.4)	1 (3.8)	0.906
ARF	3 (6.7)	3 (11.5)	0.350
Bladder injury	2 (4.4)	2 (7.7)	0.574
Ureter injury	0	1 (3.8)	0.327
Intestinal complications	1 (2.2)	1 (3.8))	0.695
Pulmonary complications	11 (24.4)	10 (38.5)	0.237
Cardiac complications	2 (4.4)	4 (15.4)	0.173
Cerebral complications	3 (6.7)	4 (15.4)	0.291
DVT	2 (4.4)	0	0.160
Sheehan syndrome	3 (6.7)	0	0.083

All data presented as mean ± standard deviation or number (%)

Shock index, heart rate / systolic blood pressure, PPH, postpartum hemorrhage; EBL, estimated blood loss; RBC, red blood cell; FFP, fresh frozen plasma; ICU, intensive care unit; DIC, disseminated intravascular coagulation; ARF, acute renal failure; DVT, deep vein thrombosis

## Discussion

The current study showed that, in patients with PPH, changes in lactic acid levels, body temperature, and SI can be important initial criteria for determining the severity of PPH and deciding whether surgical intervention is warranted. The decision for surgical intervention in patients with PPH was primarily based on EBL, blood pressure, and heart rate, in accordance with the principles of the Advanced Trauma Life Support (ATLS) classification for hypovolemic shock [18]. However, recent studies have highlighted the limitations of the ATLS classification in the management of PPH. EBL measurements can be inaccurate, and the classification may not be as sensitive for determining the optimal intervention timing in cases of PPH [19]. Some studies have shown that changes in blood lactate levels are closely associated with the prognosis of patients with septic shock [18], and higher lactic acid levels are an independent risk factor for mortality in patients with sepsis [19]. The current European guidelines on the management of major bleeding and coagulopathy following trauma suggest the use of the SI to assess the severity of hypovolemic shock [20]. A hypovolemic shock classification based on this score has been proposed (SI < 0.6: no shock; SI 0.6–1: mild shock; SI 1–1.4: moderate shock; SI ≥ 1.4: severe shock) [21]. Hypothermia is commonly accompanied by hemorrhagic shock [22]. In the present study, high lactic acid levels, low body temperature, and a high SI were all independent and significant predictors of the need for surgical intervention for PPH.

In this study, the most common cause of PPH was uterine atony alone; in multifactorial cases, the presence of accompanying atony was also a significant contributing cause of PPH. Uterine atony ranked first as the sole cause at 47.8%, and when combined with uterine atony and other causes (22.7%), the total proportion of uterine atony reached approximately 70%. This finding is consistent with previous studies revealing uterine atony as the most common cause of PPH [23]. However, after dividing the surgical intervention group into two subgroups, the proportion of cases with uterine atony and other causes in the delayed surgical intervention group increased significantly to 46.2%. It is possible that other causes, such as genital tract lacerations and remnant placental tissue, were the main causes of PPH; however, these issues were not resolved, and bleeding continued to occur over time, leading to secondary uterine atony. Placenta-related factors are known to contribute to the failure of non-surgical treatment approaches. PPH due to placenta accreta, either unanticipated or after the failure of conservative management, showed an independent and significant impact on the risk of surgical procedures [24, 25]. Additionally, an existing study showed that the leading cause of emergency peripartum hysterectomy in PPH was abnormal placentation, especially placenta accreta [26, 27]. Based on the results of this study, in cases with causes other than isolated uterine atony, especially those related to the placenta, the prognosis can be improved by considering surgical methods that can directly and quickly resolve the cause of PPH.

The subgroup analysis revealed no statistically significant differences in the initial vital signs or laboratory values between patients who underwent immediate surgery and those who underwent delayed surgery following unsuccessful non-surgical management. However, the delayed surgery group had significantly higher rates of hysterectomy and DIC. This result may be a consequence of the fact that it takes time to attempt non-surgical interventions, which may lead to a delay in performing surgery. A previous study showed that every five minutes of delay in the appropriate application of therapeutic uterotonics in patients with primary PPH resulted in an increase of 26% in the rate of hypotension and increased blood transfusions [28]. A lack of blood transfusion before surgery and prolonged surgery were also significantly associated with complications [29]. In this study, the time from transfer to surgery differed by an average of 100 min between the two subgroups. Therefore, selecting patients in whom non-surgical treatment is likely to fail and quickly deciding whether to perform surgery will help improve the prognosis of patients with PPH. Further research on this topic is required.

The subgroup analysis also showed that most complications had a higher incidence in the delayed surgical intervention group, including cerebral complications such as subdural hematoma and post-traumatic ischemia in the immediate surgery group, and ischemic stroke involving the internal carotid artery and ischemic brain damage by cardiopulmonary cerebral resuscitation in the delayed surgery group. In contrast, DVT and Sheehan syndrome had higher incidence rates in the immediate surgical intervention group. RBC transfusion and PPH were independent risk factors for postpartum thrombosis [30]. Massive blood loss over a short period may cause DVT. The pituitary gland is physiologically enlarged during pregnancy and is therefore highly sensitive to decreased blood flow caused by massive hemorrhage and hypovolemic shock. The initial insult is caused by massive PPH, which leads to impaired blood supply to the pituitary gland [31]. Therefore, DVT and Sheehan syndrome may present with signs of massive blood loss and hypovolemic shock within a short period in the immediate surgical intervention group.

This study had several limitations. Because this was a retrospective study, various variables could not be standardized; therefore, selection bias and other confounding factors may have influenced the results. Furthermore, failure of non-surgical treatment methods was defined as the failure of BBT or UAE, which are second-line treatments. However, non-surgical treatment methods for PPH are diverse, and there are limitations in establishing selection criteria because there are no clearly established treatment steps. Finally, the number of complications was small; therefore, statistical significance could not be sufficiently confirmed. Further studies with a higher number of cases are required. Despite these limitations, the main strengths of our study are that it was a large-scale study spanning over 10 years at a single institution and that meaningful results were obtained by dividing the surgery groups according to the treatment procedures performed before surgery. In addition, prognosis and postoperative complications were carefully investigated.

## Conclusions

In conclusion, our findings suggest that patients with PPH and elevated lactate levels, an increased shock index, or decreased body temperature are at a significantly higher risk of requiring surgical intervention. Additionally, uterine atony combined with other etiologies of PPH appears to be associated with a lower success rate of non-surgical management. Furthermore, delaying surgery until non-surgical interventions have failed was associated with a significantly increased risk of hysterectomy and DIC. These observations highlight the importance of identifying reliable predictors of the failure of non-surgical intervention in order to facilitate the selection of patients who might benefit from immediate surgical intervention. This approach may improve the clinical outcomes of patients with PPH.

## Data Availability

No datasets were generated or analysed during the current study.

## Abbreviations

PPH: postpartum hemorrhage
BBT: Bakri balloon tamponade
UAE: uterine arterial embolization
REBOA: Rresuscitative endovascular balloon occlusion of the aorta
EBL: estimated blood loss
BMI: body mass index
WBC: white blood cell
Hct: hematocrit
BP: blood pressure
SI: shock index
ICU: intensive care unit
RBC: red blood cell
FFP: fresh frozen plasma
DIC: disseminated intravascular coagulopathy
ARF: acute renal failure
DVT: deep vein thrombosis
ATLS: Advanced Trauma Life Support
OR: odds ratio
CI: confidence interval
